# Synchronous Multifocal Necrotizing Fasciitis in an Infant: A Case Report

**DOI:** 10.1097/INF.0000000000004900

**Published:** 2025-06-16

**Authors:** Adrien Walther, Giacomo De Marco, Oscar Vazquez, Christina Steiger, Sana Boudabbous, Romain Dayer, Dimitri Ceroni

**Affiliations:** From the * Pediatric Orthopedic Unit, Pediatric Surgery Service, Department of Woman, Child and Adolescent, Geneva University Hospitals, CH-1211, Geneva, Switzerland; † Radiology Department, Geneva University Hospitals, CH-1211, Geneva, Switzerland.

**Keywords:** Synchronous, multifocal, necrotizing fasciitis, pediatric, magnetic resonance imaging, surgery, *Streptococcus pyogenes*

## Abstract

We report an exceptional form of a synchronous multifocal necrotizing fasciitis (NF) affecting a 16-month-old girl. A high clinical suspicion of NF was confirmed by her laboratory risk indicators for necrotizing fasciitis score and magnetic resonance imaging. Blood cultures were positive for *Streptococcus pyogenes*. Successful outcomes were achieved through antibiotic treatment and an isolated surgical exploration using negative-pressure wound therapy.

Infective fasciitis—an infection of the fascia—can be necrotizing or non-necrotizing.^1^ Necrotizing fasciitis (NF), the most severe form of the disease, is defined by a rapidly progressing infection that travels along the fascial planes causing extensive muscle fascia and subcutaneous tissue necrosis. Infection typically begins in the superficial fascial planes and then progresses rapidly into the deep fascial layers. NF can be fulminant, with its systemic toxicity leading to a potentially lethal outcome if not treated promptly.^2–4^ The most common sites of infection are the limbs, accounting for approximately half of the cases,^5^ but the disease may also affect the head, neck, trunk and perineum. NF can be classified into 4 types according to their bacteriologic profile and location.^6,7^

Type I NF is polymicrobial (at least 1 anaerobic species is present in combination with 1 or more facultative anaerobic species), usually involving the trunk and perineum. Type II NF includes monomicrobial infections, usually caused by Gram-positive cocci (Group A *Streptococci* or, less frequently, *Staphylococci*) and more frequently found in the limbs. Type III NF is monomicrobial, usually involving Gram-negative species, especially from the *Vibrio* genus, and found among populations working in the fishing or aquaculture industries.^8^ Type IV NF is fungal.

Pediatric NF is much rarer and, unlike adult cases, generally occurs in otherwise healthy children who have neither a chronic disease nor any predisposition to infection.^9^ The diagnosis is primarily clinical. It is generally confirmed by surgical inspection, with dishwater-like fluid exuding from the incision and friability and necrosis of the fascia.^4^ A final confirmation should come from pathology samples.^10^

The wide variability in the initial presentation may be confusing, and differentiating a superficial infection from a deep one is often a major challenge, as is distinguishing NF from non-NF.^1,2,11^ Maintaining a high level of suspicion of NF is key, however, as the time to surgical intervention has been linked with mortality.^1,11^

There are additional means to help differentiate deep from superficial infections and NF from non-NF. Imaging often plays an important role, with ultrasound and magnetic resonance imaging (MRI) commonly used in pediatrics. The Laboratory Risk Indicator for Necrotizing Fasciitis (LRINEC) score, first proposed by Wong *et al*^11^ in 2004, is a widely used scoring system based on laboratory results that helps differentiate NF from other soft tissue infections.^11^

More recently, new concepts of how the disease spreads have been developed. It is now recognized that infection usually presents as monofocal necrotizing fasciitis (MONF). However, multifocal necrotizing fasciitis (MNF)—the development of NF at multiple noncontiguous sites—has also been reported in the literature,^12–14^ especially among elderly patients.^14^ MNF is qualified as “synchronous” when all the lesions develop within hours and as “metachronous” when they develop some days apart.^12^ These multifocal occurrences certainly contribute to diagnostic uncertainty, which could delay urgent management.

This case report aimed to describe an unusual form of synchronous MNF affecting all 4 limbs of a 16-month-old infant and to explain the imaging and laboratory tests used to reach this diagnosis.

## CASE REPORT

**FIGURE 1.**
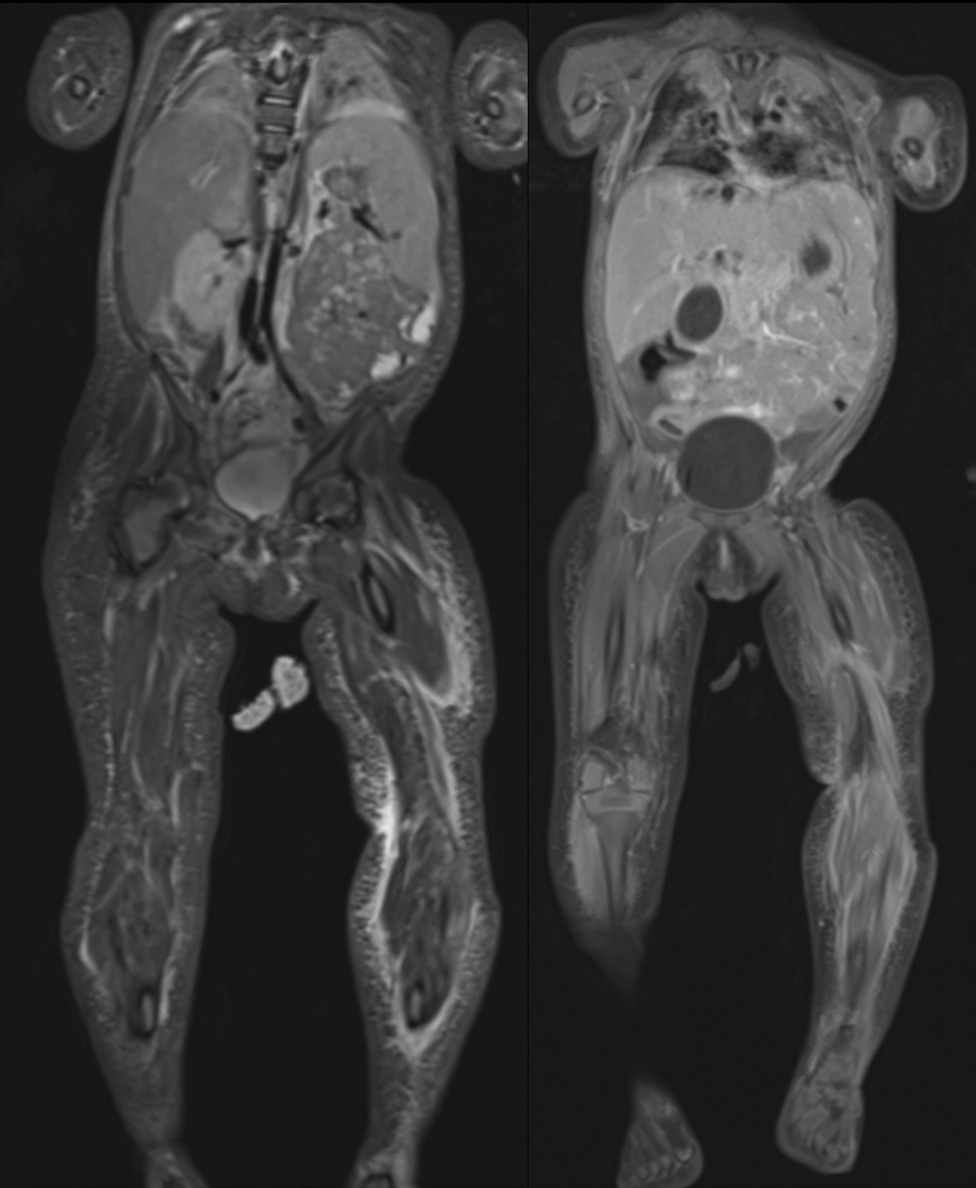
Superficial and deep multifocal fasciitis, predominantly in the left leg.

**FIGURE 2.**
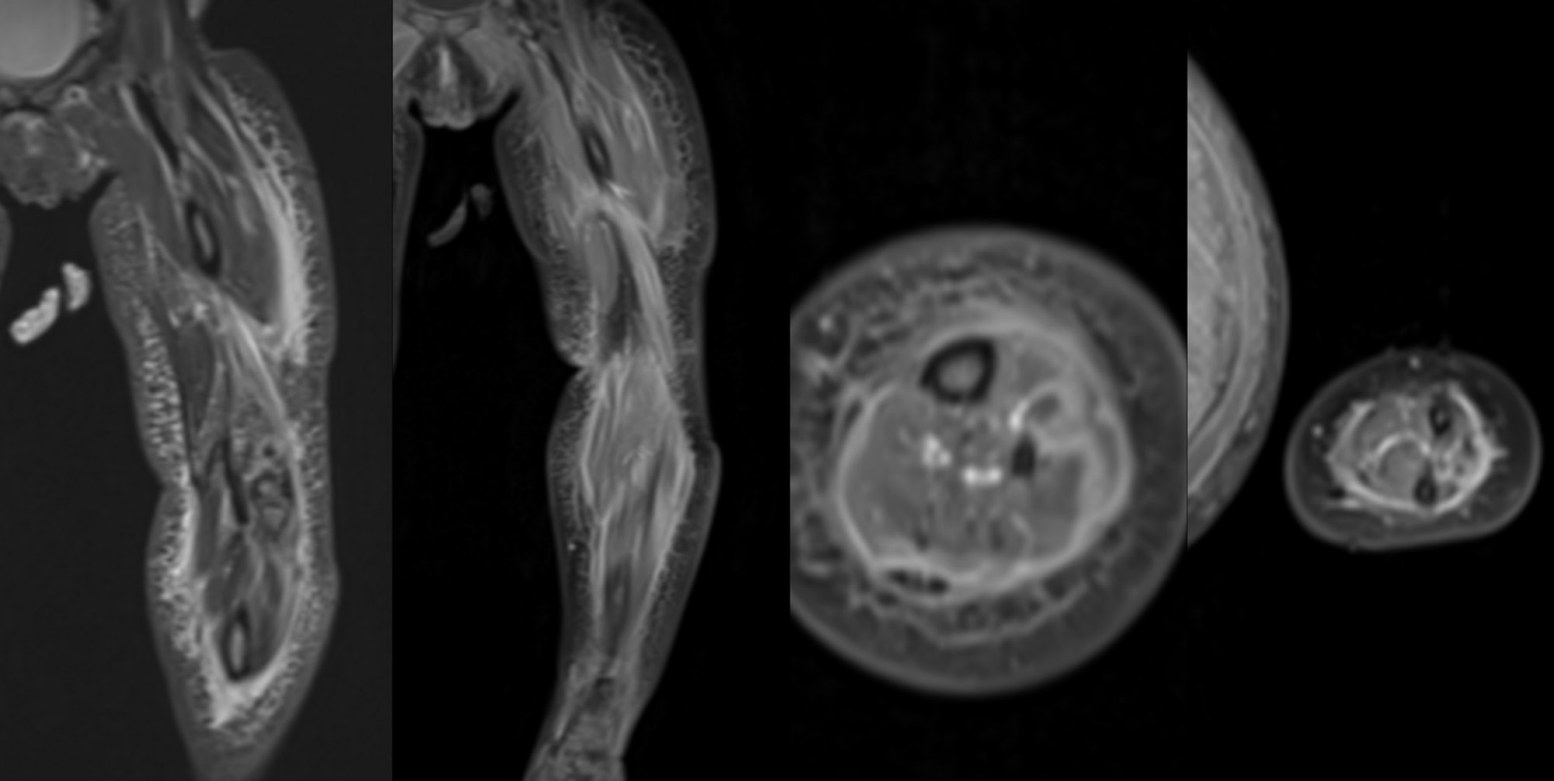
Signs of necrosis in the left leg’s toe extensor compartment (images on the left) and in the left forearm’s extensor compartment (images on the right).

A 16-month-old Caucasian girl, fully vaccinated and previously in good health, was referred to our university hospital’s pediatric emergency department by her pediatrician because of a week-long fever. The patient had suffered from acute otitis media 3 weeks before her emergency department consultation. She had been treated successfully using amoxicillin for 5 days and had recovered well. The patient had had a mild cough during fever peaks but no other signs of respiratory infection. She had suffered a single episode of vomiting during a coughing spell, with soft stools (no diarrhea) but no other digestive tract symptoms. The child was feverish at admission (38.9 °C), pale, in a weak general condition and had dark circles under her eyes. She had tachycardia, but her peripheral perfusion was maintained. Her respiratory, abdominal and neurologic statuses were within normal limits. Her throat was erythematous, with hypertrophic but exudate-free tonsils. There was no sign of otitis media, but she had multiple submandibular lymphadenopathies. Since the initial presentation suggested a fever of unknown origin, treatment was initiated using ceftriaxone. Her overall evolution during the first 48 hours was poor, and the patient developed an erythematous rash on her left upper and lower limbs, with swelling and induration of the left leg, but without crepitus, blisters or flaking. She was unable to stand, and although mobilization was unrestricted, it was painful. Laboratory results revealed an important inflammatory syndrome, with a white blood cell count of 21,000 cells/mm^3^ with a left shift, an elevated C-reactive protein level (245 mg/l) and high procalcitonin (3.27 μg/l). There was also evidence of hepatic cytolysis and an elevated level of N-terminal pro-brain natriuretic peptide (2368 ng/l) but with normal troponins and no associated organ failure. Blood gas levels on admission were within normal limits. Given this clinical evolution, suggestive of NF, the girl’s LRINEC score was calculated to be 7. As a result, the patient underwent several types of imaging examinations. Ultrasound of the soft tissues revealed no collections of fluid but some infiltration of adipose tissues. Because of her multiple osteoarticular and skin lesions, an MRI of the patient’s whole body was performed (Fig. 1), revealing signs of fasciitis in all 4 limbs that was particularly severe in the left leg. There was a thickening of the superficial and deep muscular fascia in all 4 limbs, with fluid collections in the left forearm, the left thigh, the left leg and the right leg (Fig. 2). There were also signs of myositis with intramuscular collections of fluid in the left thigh and leg. After the patient was given a first dose of ceftriaxone in the emergency department, her blood cultures came back positive for multi-sensitive *Streptococcus pyogenes*, and her antibiotic treatment was changed to intravenous amoxicillin and clindamycin. Given the clinical progression observed in the lesions during the first 48 hours of hospitalization, the results of the imaging studies and the high suspicion of NF, the patient benefited from surgical treatment during which we performed superficial and deep fasciotomies in all 4 compartments of the left leg and the medial fascia of the left thigh was also incised. Exudative fluid leaked from the incisions and was sent for culture. The subcutaneous tissue was not gray and appeared still to be bleeding slightly. The subcutaneous tissue could not easily be stripped off the superficial muscle fascia in the areas involved. The fascia appeared viable, however, and there was no skin necrosis. Widespread surgical debridement was not done, therefore, as it did not seem necessary, but intensive local washing was carried out, and extemporaneous biopsies of fascia, muscle and adipose tissue were taken. The skin incision was left open, and suction dressings were applied. Pathology examinations showed acute necrotizing inflammation of the fascia of the left thigh and the anterolateral compartment of the left leg, with bacterial pullulation only in the latter. Muscular samples taken from the left leg and left thigh revealed non-necrotizing acute inflammation. Based on all these elements and samples, the diagnosis of NF was retained. Fluid samples taken from the left leg were positive for *S. pyogenes*, while those taken from the thigh were negative. Two days after the first operation, follow-up surgery was performed, and an improvement in local status was observed with no signs of necrosis or progression. A tension-free wound closure was put in place over a deep drain that was removed 2 days later. The patient’s overall evolution was rapid and positive thereafter, enabling antibiotic therapy to continue orally using amoxicillin for 14 days after the last positive tissue sample for *S. pyogenes*.

In view of the completely atypical presentation in this case, immunologic investigations were carried out to exclude an immune deficiency. The patient’s blood count showed no leukopenia, lymphopenia or neutropenia, and immunoglobulins were within normal limits. Vaccine responses were within the norm, except for certain pneumococcal serotypes (6B, 9V, 18C and 23F, present in Prevenar 13), which were below it. Neutrophil oxidative activity was normal, as were levels of leukocyte adhesion molecules.

## DISCUSSION

We present the case of a synchronous MNF in a 16-month-old female. MNF remains an extremely uncommon condition. A 2012 literature review covering the previous 50 years identified only 33 cases.^13^ Since 2012, a further 31 patients with MNF have been described,^8,12,15–27^ bringing the total to 64 reported cases. It seems that MNF most frequently affects patients over 40 years old, which is perhaps demonstrated by the fact that only 3 patients were children, 18 months, 2 years and 3 years old.^25–27^ The case reported here involves, therefore, to the best of our knowledge, the youngest patient with MNF ever described.

This case met the definition of MNF perfectly, as defined by the occurrence of 2 or more areas of noncontiguous and noncommunicating necrosis.^13^ It is known that MNF tends to be synchronous, that is, lesions appear simultaneously during the first 24 hours. However, in some rarer situations, necrotizing lesions can develop some days after the initial one, which is known as metachronous NF.^15^ Whatever the sequence of the occurrence of the lesions, there are currently 2 theories that try to explain the development of multifocal NF. The first involves the development of metastatic septic embolization, and the second involves concurrent multifocal inoculation and the subsequent development of further NF.^18,28^

Even though the prevalence of MNF is probably underestimated and underreported, the literature suggests that the primary risk factors for MNF are linked to male sex, lower limb involvement and the type of infection. There is, however, some disagreement among authors as to the type of infections most frequently found in MNF. According to El-Khani *et al*,^13^ MNF was likely to be associated with type II disease, whereas Lee *et al*.^8^ considered that type III infections were encountered most frequently in patients with MNF and type II infections were more common among patients with MONF. Our case report and other cases of pediatric MNF suggest, therefore, that children have more type II infections. This finding is more worrying because type II NF is currently considered to be more aggressive, more able to progress and is most often associated with MNF, bacteremia and toxic shock.^13,16^

Although our patient’s infection was not fulminant, and she did not present any complications, it is crucial to highlight that patients with MNF have a much higher prevalence of bacteremia than those with MONF,^8^ at 89% and 36%, respectively. In addition, some authors have reported a higher overall mortality rate among patients with MNF than among those with MONF. Park *et al*.^29^ and Lee *et al*.^8^ reported mortality rates of 62% and 68%, respectively, in association with MNF, which are far higher rates than the 14% associated with MONF in the literature.^8^ Mortality is higher among patients who develop septic shock or toxic shock syndrome, with bandemia and thrombocytopenia considered to be strong predictors of organ failure and death.^30,31^ Indeed, these 2 parameters show that MNF rapidly exhausts the immune system and leads to overwhelming sepsis.^8^ The severity and the poor prognosis of MNF are also corroborated by the fact that there is a higher incidence of amputation among survivors.^29^

Therefore, it seems crucial to do everything possible to recognize MNF even faster, since MNF represents the more serious and widespread form of NF. Furthermore, diagnosing NF remains difficult as its typical characteristics—bullae, petechiae, skin necrosis, and crepitus—are absent in a significant number of cases.^4,32,33^ In 2004, Wong *et al*.^11^ proposed the LRINEC scoring system for distinguishing NF from other tissue infections, such as non-NF, severe cellulitis or abscesses. The score is based on 6 readily available pieces of laboratory data, usually collected at admission, such as the patient’s C-reactive protein level, white blood cell count, and hemoglobin, sodium, creatinine and glucose levels. A total score of ≥6 was initially considered highly suspicious for NF. However, there has since been some disagreement between studies as to the LRINEC’s ability to differentiate NF from severe cellulitis.^34–42^ One systematic review and meta-analysis on this subject estimated that the sensitivity and specificity of a LRINEC score ≥ 6 were only 68.2% and 84.8%, respectively.^41^ Thus, there have been many reported cases of the LRINEC score failing to diagnose NF, legitimately raising questions about its reliability because of its high false-negative rates.^37–42^ Finally, it should be remembered that the early stages of NF can give low LRINEC scores^37^ and that young children’s scores could be underestimated or falsely low.^43^

Research on NF has thus naturally turned to studies involving modern imaging techniques, with some suggesting that MRI has become the gold standard for differentiating between NF and severe cellulitis or even non-NF.^44^ MRI findings such as a thickening of the intermuscular deep fascia ≥3 mm, extensive involvement of the deep fascia, multicompartmental involvement in one limb, the presence of gas and a contrast-enhancement pattern are recognized as being suggestive of NF.^45^ A predictive scoring system has since been developed by integrating the 2 most discriminating MRI findings (ie, thickening of the intermuscular deep fascia ≥3 mm and multicompartmental involvement in one limb) with the LRINEC score for differentiating NF from severe cellulitis with non-NF.^46^ This new predictive model has better sensitivity (77% vs. 57%), positive predictive value (82% vs. 77%) and negative predictive value (79% vs. 67%) than the LRINEC score alone. In 2020, a Magnetic Resonance Indicator for necrotizing Fasciitis algorithm (the MRINEC algorithm) was developed based on a two-step decision tree including the T2 hyperintensity of intermuscular deep fascia and the diffuse T2 hyperintensity of deep peripheral fascia.^3^ The MRINEC method has better sensitivity and specificity and is becoming an indispensable method for detecting NF, especially when NF is suspected among patients with a low LRINEC score.^3^

The prognosis of a patient with NF depends on its early recognition and a prompt, aggressive surgical examination. The case reported here showed that even if the debridement of necrotic tissue was unnecessary, surgical exploration was crucial to definitively establish the diagnosis and define treatment management.

## CONCLUSIONS

This report described an exceptional case of MNF in a 16-month-old girl. Although her symptoms were initially inconclusive, they led to a high clinical suspicion of NF that was confirmed by her LRINEC score, worrying test results and MRI findings. This prompted a rapid surgical intervention and antibiotic treatment. The successful outcomes achieved by using an isolated surgical exploration and negative-pressure wound therapy were atypical, underscoring the critical importance of early detection and aggressive treatment in cases of pediatric NF.
